# Literature review and case report of post-circumcision keloid management

**DOI:** 10.1080/2090598X.2019.1651016

**Published:** 2019-08-07

**Authors:** Tim A. Buick, Wisam Abbas, Fraser D. Munro

**Affiliations:** Royal Hospital for Sick Children, Edinburgh, Scotland

**Keywords:** Keloid, penile keloid, hypertrophic scar, circumcision

## Abstract

Keloid following circumcision has been described in the literature despite the rarity of its occurrence in penile skin. In this paper, we review the literature and report the successful management of post-circumcision keloid scarring in a 2-year-old boy. After circumcision a 2-year-old boy of African origin developed keloid scarring at the circumcision site. This was treated with three intralesional injections of triamcinolone acetate over 3 months, followed by surgical excision. There was no recurrence at 6 months after excision. To our knowledge this is the 12th case of keloid following paediatric circumcision described in the literature. There is a wide range of techniques described but all are recurrence free at 6 months following repeated intralesional triamcinolone acetate injection and surgical excision. While there is no current consensus in treating post-circumcision keloid, we find that intralesional injection followed by surgical excision provides an acceptable aesthetic result, which is recurrence free.

## Introduction

Keloid scars result from an interrupted wound healing process and as such form a raised, red scar extending beyond the original wound to invade healthy skin. They are typically described as occurring in the upper chest, shoulders, upper back, and the head and neck. The ear is the most common site, accounting for just under half of all keloid scarring, closely followed by the chest and trunk [1]. The least common sites are the feet and penile skin; however, to our knowledge there have been 11 cases of penile keloid described in the paediatric literature following circumcision. As well as causing a poor aesthetic outcome, keloid scarring in this location can lead to functional disturbance. This is particularly the case if there is circumferential involvement of the circumcision site or if the scar has extended to entirely cover the glans.

## Case report

**Figure 1.**
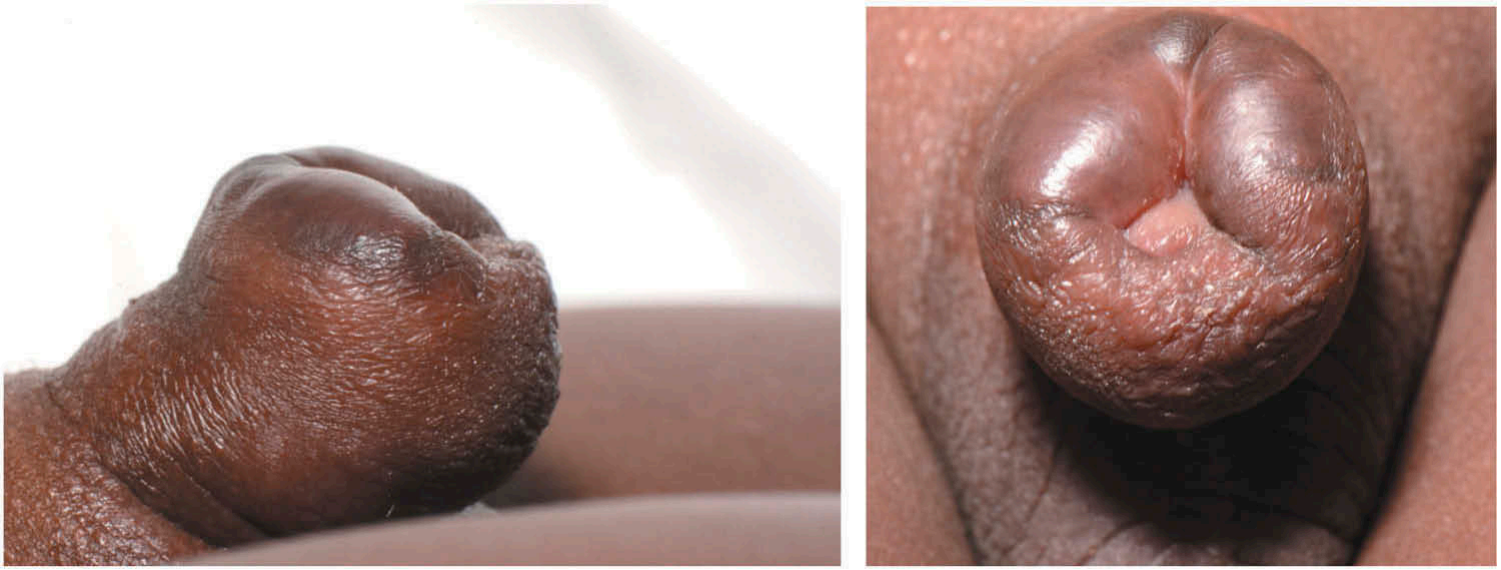
Initial presentation of keloid before any treatment. The scar is seen to extend beyond the original circumcision site up to and almost occluding the glans.

**Figure 2.**
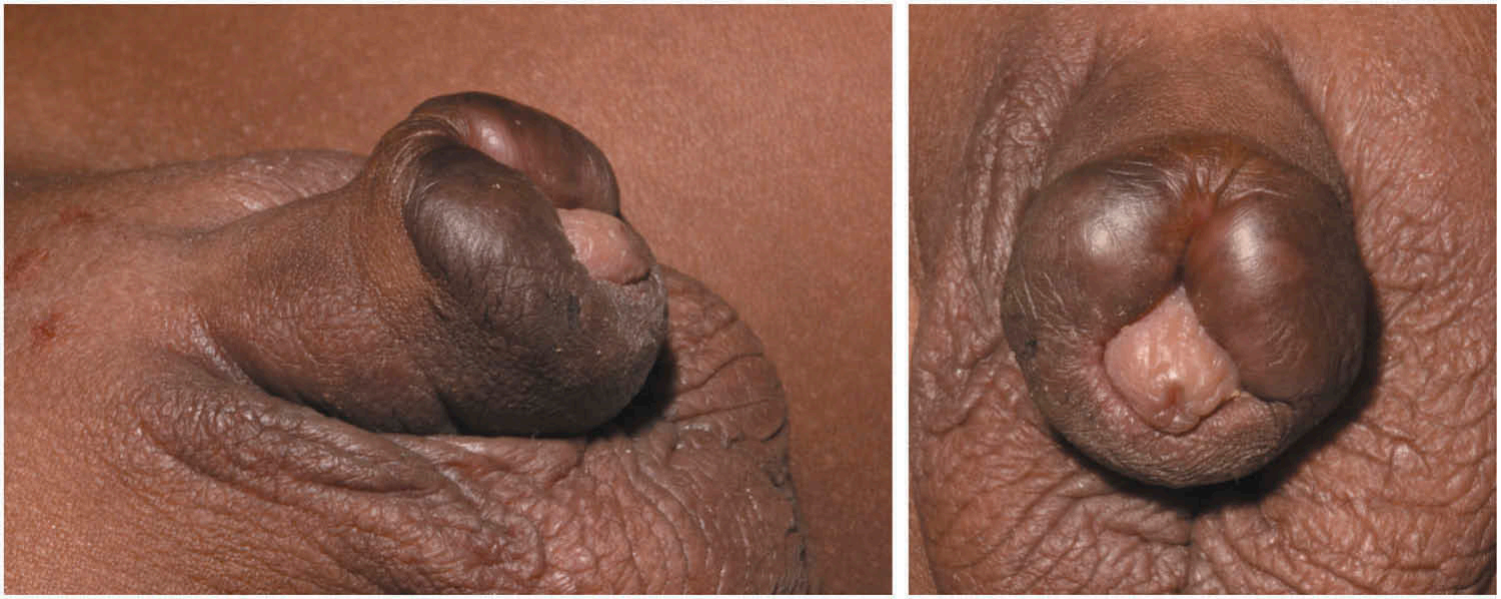
Appearance following the three injections of triamcinolone (one each month for 3 months).

**Figure 3.**
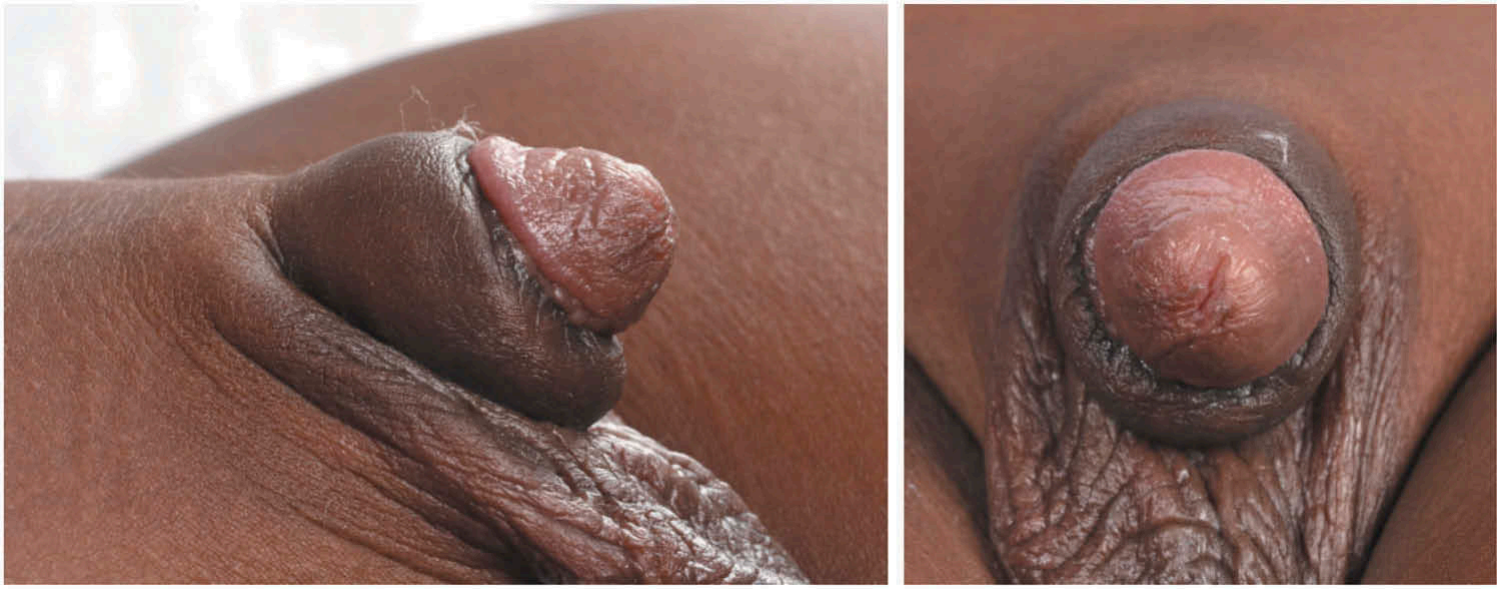
Appearance at 3 months after excision of the keloid.

A young boy of African origin underwent a religious circumcision at the age of 8 months. Around 7 months later he was referred to the paediatric surgery team by the GP with keloid scarring of the penile skin (Figure 1). This was initially treated with 1% hydrocortisone cream topically before starting a series of triamcinolone acetate injections. The keloid was injected monthly over a period of 3 months. Each time the keloid was injected with 6.5 mg triamcinolone circumferentially, and this significantly shrunk the lesion allowing surgical excision (Figure 2). The keloid was then excised 16 months after the circumcision and 1 month after the last triamcinolone injection. The outer layer of the skin was incised around the keloid tissue dorsally at the 12 o’clock position. An incision was performed between the inner layer and the keloid scar circumferentially, starting again from the dorsum. The keloid tissue was excised around the corona leaving a small sleeve of the mucosa, which was approximated to the skin with 6–0 polyglactin 910 sutures (Vicryl Rapide®; Ethicon Inc., Somerville, NJ, USA). Pathology returned dermal fibrous scarring with areas of brightly eosinophilic hyalinised collagenous tissue. The patient was discharged home on the same day and followed-up at 2 and 6 months in clinic with no recurrence and a satisfactory aesthetic result (Figure 3).

## Discussion

When keloid scarring occurs there is clearly a disruption in the normal wound healing process, which has both cellular and genetic origins. The cellular mechanism is an over expression of growth factors leading to increased fibroblast and collagen synthesis. Poorly regulated vascular endothelial growth factor, connective tissue growth factor and TGF-β, have all been implicated in keloid development [2]. The latter promotes the differentiation of fibroblasts into myofibroblasts and thus has an important role in the remodelling of granulation tissue [3]. Such growth factors become unregulated when normal wound healing is lengthened, when there is a wound infection or if there is further trauma. Other factors that predispose to keloid scarring include: increased expression of platelet-derived growth factor, interleukin-6, α1β1 integrin and Ig A, G and M [4]. However, the exact interaction between these and environmental aspects is complex.

The genetic basis of keloid remains elusive. It is more likely to occur in people of African or Asian descent [5] and there is a suggestion there is autosomal dominant inheritance [6,7]. Some rare genetic syndromes also have an increased risk of keloid formation including the short arm chromosome 16 deletion of Rubinstein–Taybi syndrome [8] and the spontaneous keloid pattern described in Goeminne syndrome [9].

Keloid of the penile foreskin was originally described by Parsons [10] in 1966 in an 8-year-old boy who had trauma to the dorsal foreskin. It was excised twice and the surrounding skin exposed to radiotherapy to prevent recurrence. Since then there have only been 11 paediatric cases occurring after circumcision described in the literature and these have been treated with a mixture of excision, triamcinolone acetate and topical steroid application, as summarised in Table 1 [11–20].

**Table 1.** A summary of paediatric case reports of penile keloid scarring post circumcision, their treatments and outcomes.

References	Patient age, years	Ethnicity	Mechanism	Treatment	Recurrence free, years
Warwick and Dickson 1993 [11]	10	Sierra Leonean	Circumcision	Serial triamcinolone injections	Not reported
Gürünlüoğlu et al. 1996 [12]	12	Caucasian	Circumcision	Serial triamcinolone injections	Not reported
Gürünlüoğlu et al. 1997 [13]	13	Caucasian	Circumcision	Serial triamcinolone injections	0.5
Bekerecioglu et al. 2005 [14]	13	Unknown	Two circumcisions	Excised and surrounding area injected with triamcinolone	1
Erdemir et al. 2006 [15]	15	Unknown	Circumcision	Excision and steroid	1
Isken et al. 2008 [16]	10	Unknown	Circumcision and prior excision	Excision and steroid	2
Demirdover et al. 2013 [17]	3	Unknown	Circumcision	Triamcinolone injection prior to excision	1
Xie et al. 2013 [18]	10	Chinese	Circumcision	Excised and surrounding area injected with triamcinolone	0.5
Xie et al. 2013 [18]	12	Unknown	Circumcision	Excised and surrounding area injected with triamcinolone	0.5
Yong et al. 2013 [19]	1.75	African	Circumcision	Excised	3
Alyami et al. 2018 [20]	13	Unknown	Circumcision	Excised and then injected with dexamethasone	3
Present study 2019	2	African	Circumcision	Triamcinolone injection prior to excision	0.5

A differential diagnosis in this case should include hypertrophic scarring, where the scar does not extend beyond the original site of trauma. Such scars often regress with time, are limited to the area of injury, have fewer thick collagen fibres (which are typically type III collagen) and scanty mucoid matrix. They also present around 1 month and show regression. In comparison, keloid scars present at ~3 months following trauma and are described as elevated and extending beyond the wound, with thick collagen (type I) and mucoid matrix [4,21,22]. In the case described above, we found a scar that extended beyond the original circumcision site, which was raised and had keloid features on pathology.

From the above literature (Table 1), none have reported recurrence of the keloid with a follow-up range of 0.5–3 years. In their case reports, Parsons [10] and Isken et al. [16] both describe patients who have previously had a lesion excised from the foreskin but no sample sent for pathology. All the other authors report specimens consistent with keloid. All described cases report a favourable outcome at least at 6 months, despite describing a wide range of surgical techniques.

Keloid formation has also been reported in penile surgery after operations for hypospadias. Recently, Alyami et al. [20] reported six cases of phalloplasty and hypospadias with one case report of a keloid forming after circumcision. When choosing how to treat these cases there are a wide range of options available. Traditionally surgical excision alone results in recurrence and so it is often augmented by other therapies [23]. These include intralesional steroid, cryosurgery, 5-flurouracil injection [24], silicone gel sheets, pressure therapy, mitomycin C, verapamil, interferon, and radiotherapy [25]. As many of these treatments are not particularly suited to penile anatomy, the most common technique used is intralesional steroid and excision.

Seven of the 11 cases in literature report no recurrence by combining intralesional triamcinolone injection with surgical excision. Triamcinolone acetate is a corticosteroid, which at a concentration of 10–40 mg/mL flattens a keloid scar by diminishing collagen synthesis and inhibiting fibroblast proliferation. In the papers listed in Table 1, triamcinolone injection intervals range from 4–6 weekly, but the optimal number of injections and duration of treatment is unclear. In the present case report, the lesion was injected monthly over a period of 3 months, which resulted in a significant reduction in the size of the keloid allowing for optimal surgical excision, with a good outcome.

## Conclusion

This case reports the successful treatment of a post-circumcision keloid scar with repeated monthly intralesional triamcinolone acetate followed by surgical excision. These are rare lesions to encounter and so optimal treatment is difficult to determine. The literature and our own experience suggest that a combination of intralesional injection and surgical excision provide the best outcome with no reported recurrence.

## References

[1] Bayat A, Arscott G, Ollier WE, et al Keloid disease: clinical relevance of single versus multiple site scars. Br J Plast Surg. 2005;58:28–37. 15629164 10.1016/j.bjps.2004.04.024

[2] Colwell AS, Phan TT, Kong W, et al Hypertrophic scar fibroblasts have increased connective tissue growth factor expression after transforming growth factor-beta stimulation. Plast Reconstr Surg. 2005;116:1387–1392. 16217483 10.1097/01.prs.0000182343.99694.28

[3] Lee TY, Chin GS, Kim WJ, et al Expression of transforming growth factor beta 1, 2, and 3 proteins in keloids. Ann Plast Surg. 1999;43:179–184. 10454326

[4] Seifert O, Mrowietz U. Keloid scarring: bench and bedside. Arch Dermatol Res. 2009;301:259–272. 19360429 10.1007/s00403-009-0952-8

[5] Chike-Obi CJ, Cole PD, Brissett AE Keloids: pathogenesis, clinical features, and management. Semin Plast Surg. 2009;23:178–184. 20676312 10.1055/s-0029-1224797 PMC2884925

[6] Marneros AG, Norris JE, Olsen BR, et al Clinical genetics of familial keloids. Arch Dermatol. 2001;137:1429–1434. 11708945 10.1001/archderm.137.11.1429

[7] Shih B, Bayat A Genetics of keloid scarring. Arch Dermatol Res. 2010;302:319–339. 20130896 10.1007/s00403-009-1014-y

[8] van de Kar AL, Houge G, Shaw AC, et al Keloids in Rubinstein-Taybi syndrome: a clinical study. Br J Dermatol. 2014;171:615–621. 25132000 10.1111/bjd.13124

[9] Fryns JP, Gevers D Goeminne syndrome (OMIM 314300): another male patient 30 years later. Genet Couns. 2003;14:109–111. 12725596

[10] Parsons RW A case of keloid of the penis. Plast Reconstr Surg. 1966;37:431–432. 5932032 10.1097/00006534-196605000-00009

[11] Warwick DJ, Dickson WA Keloid of the penis after circumcision. Postgrad Med J. 1993;69:236–237. 8497443 10.1136/pgmj.69.809.236 PMC2399721

[12] Gürünlüoğlu R, Bayramiçli M, Numanoğlu A Keloid of the penis after circumcision. Br J Plast Surg. 1996;49:425–426. 8881796 10.1016/s0007-1226(96)90018-1

[13] Gürünlüoğlu R, Bayramiçli M, Numanoğlu A Two patients with penile keloids: a review of the literature. Ann Plast Surg. 1997;39:662–665. 9418933 10.1097/00000637-199712000-00022

[14] Bekerecioglu M, Inaloz HS, Tercan M, et al Keloid formation on an inconspicuous penis. J Dermatol. 2005;32:835–838. 16361738 10.1111/j.1346-8138.2005.tb00855.x

[15] Erdemir F, Gokce O, Sanli O, et al A rare complication after circumcision: keloid of the penis. Int Urol Nephrol. 2006;38:609–611. 17111083 10.1007/s11255-006-0021-6

[16] Isken T, Sen C, Işil E, et al A very rare complication: keloid formation after circumcision, and its treatment. J Plast Reconstr Aesthet Surg. 2008;61:1405–1407. 18621597 10.1016/j.bjps.2008.02.021

[17] Demirdover C, Sahin B, Vayvada H, et al Keloid formation after circumcision and its treatment. J Pediatr Urol. 2013;9:e54–56. 22897986 10.1016/j.jpurol.2012.07.018

[18] Xie LH, Li SK, Li Q Combined treatment of penile keloid: a troublesome complication after circumcision. Asian J Androl. 2013;15:575–576. 23584379 10.1038/aja.2013.23 PMC3739232

[19] Yong M, Afshar K, Macneily A, et al Management of pediatric penile keloid. Can Urol Assoc J. 2013;7:E618–620. 24069110 10.5489/cuaj.408 PMC3776043

[20] Alyami F, Ferandez N, Koyle MA, et al Keloid formation after pediatric male genital surgeries: an uncommon and difficult problem to manage. J Pediatr Urol. 2019;15:48.e1–48.e8. 10.1016/j.jpurol.2018.08.003 30206024

[21] Jackson IT, Bhageshpur R, DiNick V, et al Investigation of recurrence rates among earlobe keloids utilizing various postoperative therapeutic modalities. Eur. J. Plast Surg.. 2001;24:88–95.

[22] Arno AI, Gauglitz GG, Barret JP, et al Up-to-date approach to manage keloids and hypertrophic scars: a useful guide. Burns. 2014;40:1255–1266. 24767715 10.1016/j.burns.2014.02.011 PMC4186912

[23] Mustoe TA, Cooter RD, Gold MH, et al International clinical recommendations on scar management. Plast Reconstr Surg. 2002;110:560–571. 12142678 10.1097/00006534-200208000-00031

[24] Sadeghinia A, Sadeghinia S Comparison of the efficacy of intralesional triamcinolone acetonide and 5-fluorouracil tattooing for the treatment of keloids. Dermatol Surg. 2012;38:104–109. 22093096 10.1111/j.1524-4725.2011.02137.x

[25] Durani P, Bayat A Levels of evidence for the treatment of keloid disease. J Plast Reconstr Aesthet Surg. 2008;61:4–17. 17644502 10.1016/j.bjps.2007.05.007

